# Factors for successful implementation of population-based expanded carrier screening: learning from existing initiatives

**DOI:** 10.1093/eurpub/ckw110

**Published:** 2016-08-01

**Authors:** Kim C.A. Holtkamp, Inge B. Mathijssen, Phillis Lakeman, Merel C. van Maarle, Wybo J. Dondorp, Lidewij Henneman, Martina C. Cornel

**Affiliations:** 1 Department of Clinical Genetics, Section of Community Genetics and EMGO Institute for Health and Care Research, VU University Medical Center, Amsterdam, The Netherlands; 2 Department of Clinical Genetics, Academic Medical Center, Amsterdam, The Netherlands; 3 Department of Health, Ethics & Society, Research Institutes CAPHRI and GROW, Maastricht University, Maastricht, The Netherlands

## Abstract

**Background:** Carrier screening for autosomal recessive disorders aims to facilitate reproductive decision-making by identifying couples with a 1-in-4 risk in every pregnancy of having an affected child. Except for a few countries or regions, carrier screening is not widely offered and is mostly ancestry-based. Technological advances enable carrier screening for multiple diseases simultaneously allowing universal screening regardless of ancestry (population-based expanded carrier screening). It is important to study how this can be successfully implemented. This study therefore aims to identify critical factors involved in successful implementation, from a user perspective, by learning from already implemented initiatives. **Methods:** Factors associated with successful implementation were identified by: (i) a literature review and (ii) two case studies; studying experiences with carrier screening in two high-risk communities (a Dutch founder population and the Ashkenazi Jewish population), including a survey among community members. **Results:** Factors identified were familiarity with (specific) genetic diseases and its availability, high perceived benefits of screening (e.g. screening avoids much suffering), acceptance of reproductive options, perceived risk of being a carrier and low perceived social barriers (e.g. stigmatization). In contrast to the Jewish community, the initial demand for screening in the Dutch founder population did not entirely come from the community itself. However, the large social cohesion of the community facilitated the implementation process. **Conclusion:** To ensure successful implementation of population-based expanded carrier screening, efforts should be made to increase knowledge about genetic diseases, create awareness and address personal benefits of screening in a non-directive way.

## Introduction

When both partners are carriers of the same autosomal recessive (AR) disease, they have a 1-in-4 risk of having an affected child in each pregnancy. An estimated 1–2 of every 100 couples in the general population face such a risk.^1^ These include couples who have a higher risk based on their ancestral background. Carrier screening allows couples to find out whether both partners are carriers, and if so, facilitates informed reproductive decision-making, including accepting this risk or refraining from having children, prenatal diagnosis (PND), preimplantation genetic diagnosis (PGD), using donor gametes, adoption or trying to find another partner.

Carrier screening for AR diseases has only been realized in a few countries or communities and is mostly ancestry-based. The Ashkenazi Jewish (AJ) community, for example has been familiar with screening for Tay-Sachs Disease (TSD) since the 1970s.^2^ In Cyprus, carrier screening for β-thalassaemia is a well-known phenomenon that has led to a significant decrease in its birth prevalence.^3^ In the United States, carrier screening for cystic fibrosis (CF) is offered preconceptionally or early in pregnancy, regardless of ancestry.^4^

In the Netherlands and elsewhere, both the target population (i.e. couples planning a pregnancy) and health professionals showed positive attitudes regarding (preconception) carrier screening for CF and haemoglobinopathies (HbPs).^5–7^ Health professionals also express concerns for example time constraints and a need for education.^8^ In 2007, the Health Council of the Netherlands recommended studying preconception carrier screening for CF and HbPs in a large-scale nation-wide pilot study. Although this pilot was not realized, some local initiatives did succeed in implementing carrier screening within healthcare.

Due to ongoing technological developments (next generation sequencing), it is now possible to screen for multiple AR diseases simultaneously. So-called expanded carrier screening panels are currently being developed and offered, mainly commercially. These panels allow screening regardless of ancestry or geographical origin (population-based or universal) and will increase equity of access and reduce stigmatization.^9^^,^^10^ Inevitably, such panels comprise diseases or genetic variants which do not pose a high risk for all members of the target group. In this study, we aim to identify factors for successful and responsible implementation of population-based expanded carrier screening. An essential aspect is to specify the aim of such screening. Although in some communities with a high disease burden, prevention of the birth of an affected child may be an explicit and well-accepted purpose of screening,^3^^,^^11^ providing couples with meaningful options for autonomous reproductive choice is considered the main aim.^11^ It is widely accepted that this should also be the aim of population-based expanded carrier screening.^10^ The question then is how can population-based expanded carrier screening with this specific aim be successfully and responsibly implemented, and what can be learned from existing initiatives?

## Methods

Most theoretical frameworks for implementation, for example the framework developed by Grol and Wensing,^12^ study factors on different levels (e.g. structural, organizational, provider and innovation level) emphasizing the role of professionals in this process. However, as Achterbergh et al.^13^ showed not only professionals (e.g. scientists, policy makers, health professionals) but also citizens/users have an inevitable impact on the implementation process. In this article, we therefore especially address the user perspective.

### Literature and case studies

We identified critical factors for successful implementation of carrier screening from a user perspective by using mixed methods. Data collection involved obtaining a general overview of factors described in the literature. Between January 2014 and January 2016, PubMed was searched for relevant peer-reviewed articles describing the implementation and uptake of carrier screening programmes (e.g. for CF, HbPs, Jewish community). Additional articles were identified by citation searching of the references of selected articles. Recurring factors for successful implementation identified were then compared with factors found in case studies in two communities that have already experienced successful implementation of (ancestry-based) carrier screening. The first case study (Box 1) concerned a Dutch founder population where carrier screening for four, generally rare and severe, AR diseases has been offered via an outpatient clinic since 2012.^14^ The second case study (Box 2) was the AJ community, where, worldwide, carrier screening has been offered for decades and where screening panels have expanded over the years. Based on the findings of the literature search, a questionnaire was developed by a multidisciplinary research team (clinical geneticists, health scientists and an ethicist) and distributed in these communities in 2014.

Box 1 Carrier screening in a Dutch founder population—Case study 1

Since 2012, carrier screening for four severe AR diseases has been offered via a preconception care consultation (special outpatient clinic) to inhabitants of a genetic isolate in the North-Western part of the Netherlands.^14^ This Roman Catholic village was founded in the fourteenth century by 7–20 families and due to religious and social factors, this village has a large social cohesion. As a result of common ancestral origin, certain severe genetic diseases that are generally very rare are much more frequent in this population due to the presence of so-called founder mutations. The four diseases are pontocerebellar hypoplasia type 2 (PCH2), a specific genetic form of foetal akinesia deformation sequence (FADS), rhizomelic chondrodysplasia punctate type 1 (RCDP1) and osteogenesis imperfecta (OI) type IIB/III.^14^ Furthermore, other (non-lethal) diseases are also highly frequent in this community. Of all individuals tested in the first year of the outpatient clinic, 1 in 3 was identified as being a carrier of at least one disease and four carrier couples were identified.^14^

Box 2 Carrier screening in the AJ community—Case study 2

The AJ community worldwide is at risk for several severe genetic diseases due to genetic drift and founder effect. In the 1970s, carrier screening for TSD was introduced from within the community itself.^2^ Carrier screening has now expanded to many more diseases. The American College of Medical Genetics, for example, recommends screening for eight diseases in this population.^15^ In 2015, a carrier screening panel for nine severe hereditary diseases (TSD, Familial dysautonomia, Canavan disease, Bloom syndrome, Fanconi Anaemia group C, Glycogen Storage disease type 1a, Mucolipidosis type IV, Niemann-Pick type A and CF) has been developed by the Academic Medical Center in Amsterdam and is available in university hospitals in the Netherlands, with Gaucher disease as 10th optional disease, due to its less severe character. Though the uptake of carrier screening in the Jewish community worldwide is generally high,^16^^,^^17^ this is, up to now, not the case in the Netherlands.

### Questionnaire: participants and procedure

Individuals from the Dutch founder population (*n* = 600) were recruited by two general practitioners (GPs), who each randomly selected 200 men and 200 women of reproductive age (18–40 years), and 100 men and 100 women between 40 and 70 years from two different post code areas. Questionnaires were sent to people’s home address accompanied by a letter from their GP including background information about the study. The overall response rate was 34%, resulting in a sample of 206 respondents.

Individuals from the Dutch AJ community were invited to complete a similar questionnaire. Details are published elsewhere.^18^ No response rates could be calculated here because data collection was performed online; 145 respondents were included in the final analyses.^18^ The Medical Ethical Committee of Academic Medical Center Amsterdam and VU University Medical Center Amsterdam approved both study protocols.

### Survey instrument

The questions that were asked were similar for both communities, although there were some differences related to community-specific characteristics (e.g. different diseases and specific options as ‘rabbi’ and ‘Dor Yeshorim’ for the AJ community). Familiarity with genetic diseases and carrier screening was measured by means of two questions: ‘do you or your partner know someone with a severe genetic disease?’ (if yes, who and what disease) and ‘have you ever heard of a carrier test for diseases relatively common in [your community]?’ Attitude towards the offer of carrier tests was measured using a semantic differential five-point scale with four bipolar adjective pairs: good–bad, alarming–not alarming, desirable–not desirable, self-evident–not self-evident. Additionally, respondents were asked to indicate the extent to which they agreed with three statements regarding perceived benefits (e.g. offering carrier tests avoids suffering), perceived social barriers (e.g. offering a carrier test leads to anxiety in the community), the acceptability of reproductive options (e.g. PND, termination of pregnancy and PGD), one statement regarding partner choice (i.e. carrier test results can help when choosing a partner) and one regarding perceived risk of being a carrier. All items were answered on a five-point scale [strongly disagree (1) to strongly agree (5)]. Respondents also indicated their preference for an ancestry-based panel, specifically aimed at their community or a population-based expanded panel that is offered regardless of ancestry.

Finally, socio-demographic data including gender, age, level of education, religiousness, marital status, having children and planning to have children were collected (Supplementary Table S1).

### Data preparation and analysis

Descriptive analyses were used for respondents’ characteristics. Principle factor analysis with varimax rotation was used to assess possible subscales in the questions measuring attitude, perceived benefits, perceived social barriers and acceptability of reproductive options. Reliability analysis for internal consistency of the scales was performed. Univariate and multiple logistic regression analyses were used to identify possible factors associated with a positive attitude (≥4, range 1–5) towards carrier screening. Scale scores were dichotomized by the median. Statistical significance was set at *P* < 0.05 (*P <* 0.10 for the univariate analysis). All analyses were performed using IBM SPSS version 20 for Windows (IBM Corp, Armonk, NY, USA).

## Results

Both the literature and the case studies identified several critical factors for successful implementation of carrier screening from a user perspective: familiarity with genetic diseases and (the availability of) carrier screening, perceived benefits of carrier screening, perceived risk, social influences and community support (Table 1). Supplementary Tables S1–S4 present the results from the questionnaire study.

**Table 1:** Factors identified of being of importance for the successful implementation of carrier screening from a user perspective

	People from a Dutch founder population	People from the Dutch Jewish community
Familiarity		
Familiarity with genetic diseases	High^a^	Medium^b^
Familiarity with genetic carrier screening	High	Medium
Perceived benefits		
Perceived benefits of genetic carrier screening	High	High
Acceptance of reproductive options	High	High
Perceived risk		
Perceived risk of being a carrier	Medium	Medium
Social influences		
Perceived (social) barriers of genetic carrier screening	Low^c^	Low
Community support	High	High

^a^High: factor highly present in community.

^b^Medium: factor somewhat present in community.

^c^Low: factor not/less present in community.

### Familiarity

The implementation literature stresses that knowledge of and familiarity with the problem addressed by an innovation among the public is important in the adoption, diffusion and dissemination of innovations.^19^^,^^20^ The process of learning new information is furthermore shaped by prior experiences.^21^ Regarding carrier screening, prior experiences with genetic diseases, illness and disability may play a pivotal role in thinking about the value of reproductive tests and decision-making.^20^,^22^ The two case studies indicate that people within these communities are relatively familiar with genetic diseases. Sixty-two percent of the respondents from the founder population and 41% from the Jewish community knew someone with a severe genetic disease. Furthermore, 82% of the founder population and 65% of the Jewish community had heard about carrier screening.

### Perceived benefits

Positive attitudes towards carrier screening among the public have been shown.^3^^,^^5^^,^^6^^,^^23^ For example, the vast majority of the general population favours the availability of a routine offer of CF carrier screening.^7^ In a review on factors affecting decisions to accept or decline CF carrier screening, it was found that in 35% of the studied articles ‘high perceived benefits of screening’ positively influenced the decision to accept screening.^24^ Similar results were found in our two cases. Both respondents from the founder population and from the Jewish community were highly positive about carrier screening in their community [Mean (*M*) = 4.26 and *M* = 4.14, range 1–5, respectively] and perceive high benefits (*M* = 4.44 and *M* = 4.23, range 1–5). Respondents reporting high perceived benefits more often had a positive attitude towards carrier screening (Supplementary Tables S3 and S4). Respondents in both communities slightly preferred a population-based expanded offer that screens for a wide array of diseases and is not aimed solely at specific high risk groups (preferred by 51.3 and 53.8%, respectively) instead of ancestry-based carrier screening (preferred by 44.7 and 42.8%, respectively).^18^ In both communities, the most important reason for preferring population-based expanded screening was that ‘everyone has a right to be tested’ [37.7% (Dutch founder population) and 32.1%^18^ (AJ community)]. ‘Prevention of high healthcare costs’ was mentioned as an important reason against population-expanded screening among those in favour of ancestry-based screening (19.6 and 33.9%,^18^ respectively), and 27% in both groups indicated that ‘screening should better be based on high risk’.

As described by Laberge et al.,^25^ consensus in favour of avoiding affected births is one of the factors involved in the success of TSD and β-thalassaemia carrier screening programmes. This consensus would seem crucial for the successful implementation of programmes in which reducing birth prevalence is the explicit aim. However, also for programmes that primarily aim to improve reproductive autonomy, it is important that the reproductive choices made available by the screening offer (including those that allow couples to avoid the birth of an affected child) are recognized as meaningful by the target group. Both the founder population and the Jewish community showed a relatively high acceptance of reproductive options such as PND (*M* = 4.61 and *M* = 4.28) and PGD (*M* = 4.35 and *M* = 4.12). Termination of the pregnancy in case of an affected foetus was also considered acceptable (*M* = 4.10 and *M* = 3.67, respectively). With respect to the influence of carrier screening on partner choice, people from the Jewish community (*M* = 2.59, range 1–5) more often agreed that carrier test results can help when choosing a partner than people from the founder population (*M* = 1.67, range 1–5) (Supplementary Table S2).

### Perceived risk

An aspect contributing to a positive implementation climate for an innovation is ‘compatibility’.^19^ This comprises the extent to which meaning and values attached to an innovation correspond with, for example, individuals’ own norms, values and perceived risks.^19^ In several behaviour change theories (e.g. Health Belief Model), perceived risk is assigned a role in predicting (health) behaviour.^26^ The actual effects of perceived risk on behaviour are questioned as some argue that excessive worry or perceived risk inhibits screening behaviour, while others show that a certain degree of perceived risk ensures motivation.^27^^,28^ This contrast is also present in the field of carrier screening. Some studies confirmed that the perceived risk of passing on a recessive disease or being a carrier positively influenced intention or decision to participate in carrier screening,^29^^,^^30^ while others did not show this relation.^31^ In our study, respondents from both the founder population (*M* = 2.94, range 1–5) and the Jewish community (*M* = 2.59, range 1–5) are, to some extent, worried about their risk of being a carrier, but a significant relation between perceived risk and, in this case, a positive attitude towards carrier screening could not be confirmed.

### Social influences

A weaker perception of (social) barriers, like perceiving negative consequences from testing such as social stigma, affects decisions to accept carrier screening.^24^ In the context of HbP carrier screening, fear of social stigmatization impeded its successful implementation in the past.^32^ Our two case studies showed that respondents from the founder population and the Dutch Jewish community both perceived low social barriers to carrier screening (*M* = 2.00 and *M* = 2.27, range 1–5), which correlated with a positive attitude towards screening.

### Community support

Successful implementation of TSD screening is partly due to the perceived severity of the disease among the Jewish community, and the involvement of clinicians and community leaders in the development of carrier screening programmes.^25^ Carrier screening programmes for the Jewish community are furthermore often funded by the population itself.^33^ In the Netherlands, carrier screening for the AJ community is available in university hospitals but is not often requested. As our earlier research has shown, most individuals of AJ descent, who had screening, bought these tests abroad,^18^ and little use is made of the local offer, most likely due to unawareness of the community regarding its availability. In the Dutch founder population, screening was first advocated by clinical geneticists in response to questions from the community resulting in awareness among primary care professionals and later among the community itself. The strong social cohesion of this community furthermore facilitated the process of implementing screening.

## Discussion

Various factors from a user perspective seem to support successful implementation of carrier screening: high familiarity with genetic diseases, the availability of carrier testing, high perceived benefits, acceptance of reproductive options, perceived risk of being a carrier, low perceived social barriers and community support.

The two communities in which ancestry-based carrier screening was implemented successfully showed high familiarity with genetic diseases and genetic carrier testing compared with the general population. In a survey among the general population in the Netherlands, one-third reported that they knew someone with a hereditary disease.^34^ Diseases mentioned, however, were mostly multifactorial, like cardiovascular diseases and asthma, and it can therefore be expected that familiarity is lower for AR diseases.

Our results showed that both communities perceive high personal benefits of screening. Theoretical models regarding behaviour change (e.g. Health Belief Model) have described the influence of perceived benefits on one’s intention, attitude and eventually one’s behaviour.^28^ In their review on factors affecting the decision to accept or decline CF carrier screening, Chen and Goodson^24^ show that people who perceived high benefits were more likely to accept screening, whereas the lack of a recognized rationale for screening (testing without increased risk) can act as a barrier.^35^^,36^

The stimulating role of key figures (e.g. people who have personal experience with carrier screening and religious leaders) within a community is essential in empowering the public.^19^^,37^^,38^ The two populations described here are unique examples when it comes to community support. In the Jewish community, especially internationally, carrier screening was initiated by the community itself and supported by respected community leaders (e.g. rabbis and physicians).^2^^,^^39^ A bottom-up approach emerged where the problem is first defined by the target community and where community members experience high ownership. The founder population, however, showed that even when the demand for screening does not initially come from the community itself, community support facilitates successful implementation as well.

For population-based expanded carrier screening however, the presence of the critical factors as identified is questionable. The general population is expected to be less familiar with carrier screening and genetic diseases, the perceived risk of being a carrier is likely to be absent, and generally, people are expected to be unaware of the possible personal benefits of screening. Furthermore, when implementing population-based expanded carrier screening, the stimulating role of community support is likely to be less evident, as there is no specific community with which people can identify themselves. Given the primary aim of screening, (increasing autonomous reproductive decision-making) what does the absence of those factors mean in the context of successful implementation of population-based expanded carrier screening? Do people perceive an offer of carrier screening as meaningful? And if not, should we continue offering?

The lack of familiarity, due to the character of AR diseases, can cause people to be unaware of possible increased risks and possible advantages that screening may have. If an estimated 1–2% of all couples is a carrier couple of an AR disease, which is higher than the general risk for foetal aneuploidy, population-based expanded carrier screening might indeed be meaningful for prospective parents. Whether people actually perceive this offer as meaningful should be studied. Since the current screening offers of population-based expanded carrier screening are mainly technology-driven, it is important to attune to the actual demand. In discussing the responsible implementation of population-based expanded carrier screening as well as in identifying whether there is an actual demand for screening, an active role should be adopted by governments and public health authorities.^10^ Considering the primary aim of screening however, a high uptake is not a criterion for success just as it is the case for prenatal screening for foetal aneuploidy.^40^

By using two examples of communities where carrier screening has been quite successfully implemented, this article provides a unique insight into possible critical factors for implementing screening on a population level. Another strength is its focus on a user perspective, which is often less extensively discussed. However, more factors might influence successful implementation (e.g. costs and reimbursement of screening). To create a complete overview of the implementation process, it is important to study all stakeholders and the entire process, and incorporate existing implementation theories.

In conclusion, the lessons learned show that most of the critical factors for successful implementation of carrier screening are less evident when it comes to population-based expanded carrier screening. Furthermore, to achieve the primary aim of carrier screening, increasing couple’s reproductive autonomy, it is important that people consider carrier screening as being meaningful. Only then can screening be implemented responsibly. To further develop population-based expanded carrier screening responsibly, effort should be made to increase knowledge about genetic diseases, create awareness, facilitate public debate about the pros and cons of screening and address personal benefits of screening in a non-directive way.

## Supplementary Material

Supplementary Data
