# Supplementary material for: Factors for successful implementation of population-based expanded carrier screening: learning from existing initiatives
Source: Eur J Public Health. 2016 Aug 1;27(2):372–7. doi: 10.1093/eurpub/ckw110 (PMC5421354; doi:10.1093/eurpub/ckw110)
Supplement: Supplementary Data [file ckw110_Supplementary_Data.zip › ejph-2016-03-om-0188-File006.docx]

| Items | People from a Dutch founder population |  | People from the Dutch Jewish community |
| --- | --- | --- | --- |
|  | **(*N*=206)**  **Mean (SD)^a^** |  | **(*N*=145)**  **Mean (SD)^a^** |
| Attitude towards carrier screening^b^ | 4.14 (.84) |  | 4.26 (.58) |
| Perceived benefits^c^ | 4.44 (.67) |  | 4.23 (.77) |
| Acceptability of reproductive options^d^ | 4.35 (.73) |  | 4.03 (.98) |
| Partner choice | 1.67 (1.12) |  | 2.59 (1.37) |
| Perceived risk | 2.94 (1.46) |  | 2.59 (1.33) |
| Perceived social barriers^e^ | 2.00 (.82) |  | 2.27 (.88) |

**Table S2** Mean scores and standard deviations (SD) of both the Dutch founder population and the Dutch Jewish community on the different subscales and single items

^a^The higher the mean score, the higher the agreement.

^b^Cronbach’s α: 0.64.

^c^Cronbach’s α: 0.70.

^d^Cronbach’s α: 0.74.

^e^Cronbach’s α: 0.62
