# Supplementary material for: Factors for successful implementation of population-based expanded carrier screening: learning from existing initiatives
Source: Eur J Public Health. 2016 Aug 1;27(2):372–7. doi: 10.1093/eurpub/ckw110 (PMC5421354; doi:10.1093/eurpub/ckw110)
Supplement: Supplementary Data [file ckw110_Supplementary_Data.zip › ejph-2016-03-om-0188-File007.docx]

**Table S3** Univariate and multiple logistic regression: factors associated with a positive attitude towards carrier screening in the Dutch founder population

| **Variable** | **Univariate** | |  | **Multiple** | |  |
| --- | --- | --- | --- | --- | --- | --- |
|  | **Positive attitude**  **(*N*=141)** | |  | **Positive attitude**  **(*N*=141)** | |  |
|  | **OR^a^** | **(95%CI^b^)** | ***P* value** | **OR** | **(95%CI)** | ***P v*alue** |
| Age | 0.86 | (0.40-1.84) | 0.694 | **-** | **-** | **-** |
| Level of education  Low^c^  Medium  High | 1.15  0.98 | (0.51-2.58)  (0.43-2.24) | 0.734  0.957 | **-**  **-** | **-**  **-** | **-**  **-** |
| Religious activity | 1.06 | (0.81-1.39) | 0.676 | **-** | **-** | **-** |
| Planning to have (more) children | 0.81 | (0.41-1.57) | 0.524 | **-** | **-** | **-** |
| Familiarity with genetic disease | 2.00 | (1.00-3.87) | **0.054** | 2.30 | (0.99-5.33) | 0.053 |
| Familiarity with carrier screening | 1.32 | (0.56-3.11) | 0.531 | **-** | **-** | **-** |
| High perceived benefits | 2.07 | (1.06-4.06) | **0.034** | 1.43 | (0.59-3.47) | 0.431 |
| High acceptability of reproductive options | 3.03 | (1.40-6.56) | **0.005** | 2.50 | (1.10-5.66) | **0.029** |
| High perceived risk | 1.27 | (0.64-2.53) | 0.490 | **-** | **-** | **-** |
| Low perceived social barriers | 4.46 | (2.20-9.04) | **<0.001** | 4.71 | (2.07-10.73) | **<0.001** |

^a^Odds ratio (OR)

^b^Confidence interval (CI)

^c^Reference category
