# Supplementary material for: Factors for successful implementation of population-based expanded carrier screening: learning from existing initiatives
Source: Eur J Public Health. 2016 Aug 1;27(2):372–7. doi: 10.1093/eurpub/ckw110 (PMC5421354; doi:10.1093/eurpub/ckw110)
Supplement: Supplementary Data [file ckw110_Supplementary_Data.zip › ejph-2016-03-om-0188-File008.docx]

**Table S4** Univariate and multiple logistic regression: factors associated with a positive attitude towards carrier screening in the Ashkenazi Jewish community

| **Variable** | **Univariate** | |  | **Multiple** | |  |
| --- | --- | --- | --- | --- | --- | --- |
|  | **Positive attitude**  **(*N*=95)** | |  | **Positive attitude**  **(*N*=95)** | |  |
|  | **OR^a^** | **(95%CI^b^)** | ***P* value** | **OR** | **(95%CI)** | ***P* value** |
| Age | 1.33 | (0.67-2.68) | 0.417 | **-** | **-** | **-** |
| Level of education  Low^c^  Medium  High | 0.42  1.17 | (0.34-5.31)  (0.10-13.34) | 0.505  0.899 | **-**  **-** | **-**  **-** | **-**  **-** |
| Religious activity | 0.44 | (0.14-1.41) | 0.169 | **-** | **-** | **-** |
| Planning to have (more) children | 0.92 | (0.42-2.03) | 0.843 | **-** | **-** | **-** |
| Familiarity with genetic disease | 1.92 | (0.93-3.97) | **0.080** | 1.23 | (0.49-3.10) | 0.664 |
| Familiarity with carrier screening | 1.88 | (0.93-3.84) | **0.082** | 1.51 | (0.61-3.79) | 0.375 |
| High perceived benefits | 8.61 | (3.94-18.83) | **<0.001** | 6.44 | (2.72-15.20) | **<0.001** |
| High acceptability of reproductive options | 3.59 | (1.74-7.41) | **0.001** | 3.26 | (1.37-7.74) | **0.007** |
| High perceived risk | 1.73 | (0.78-3.82) | 0.473 | **-** | **-** | **-** |
| Low perceived social barriers | 6.30 | (2.86-13.89) | **<0.001** | 5.31 | (2.18-12.95) | **<0.001** |

^a^Odd ratio (OR)

^b^Confidence interval (CI)

^c^Reference category
