# Supplementary material for: Factors for successful implementation of population-based expanded carrier screening: learning from existing initiatives
Source: Eur J Public Health. 2016 Aug 1;27(2):372–7. doi: 10.1093/eurpub/ckw110 (PMC5421354; doi:10.1093/eurpub/ckw110)
Supplement: Supplementary Data [file ckw110_Supplementary_Data.zip › ejph-2016-03-om-0188-File005.docx]

**Table S1** Characteristics of respondents

|  | **People from a Dutch founder population**  **(*N*=206)** | **People from the Dutch Jewish community^a^**  **(*N*= 145)** |
| --- | --- | --- |
|  | ***N* (%)** | ***N* (%)** |
| Gender |  |  |
| Male | 68 (33.0) | 43 (29.7) |
| Female | 138 (67.0) | 102 (70.3) |
| Age (years) |  |  |
| 18-45 | 149 (72.3) | 79 (54.5) |
| ≥46 | 57 (27.7) | 66 (45.5) |
| Education^b^ |  |  |
| Low | 68 (33.2) | 3 (2.1) |
| Intermediate | 78 (38.0) | 24 (16.7) |
| High | 59 (28.8) | 117 (81.3) |
| Religious activity |  |  |
| Very/somewhat active | 67 (33.5) | 125 (86.2) |
| Not active/not applicable | 133 (66.5) | 20 (13.8) |
| Marital status |  |  |
| Single | 40 (19.4) | 41 (28.3) |
| Cohabiting/married | 164 (79.6) | 98 (67.6) |
| Divorced/widowed | 2 (1.0) | 2 (1.4) |
| Other^c^ |  | 4 (2.8) |
| Having children |  |  |
| Yes | 124 (60.2) | 97 (66.9) |
| No | 82 (39.8) | 48 (33.1) |
| Planning to have (more) children^d^ |  |  |
| Yes/Maybe | 98 (47.8) | 53 (47.4) |
| No/Not applicable | 107 (52.2) | 59 (52.6) |
| Familiarity with genetic disease |  |  |
| Yes | 127 (62.6) | 59 (40.7) |
| No | 76 (37.4) | 86 (59.3) |
| Familiarity with carrier screening |  |  |
| Yes | 170 (82.9) | 94 (64.8) |
| No | 35 (17.1) | 51 (35.2) |

^a^Data in this column have also partly been published in Holtkamp et al.^15^

^b^Low: primary school, lower level of secondary school, lower vocational training. Intermediate: higher level of secondary school, intermediate vocational training. High: higher vocational training, university.

^c^Other includes: engaged, non-cohabiting but with partner.

^d^Data for the Dutch Jewish community only available for 112 respondents having a partner.
